# Biomechanical changes following corneal crosslinking in keratoconus patients

**DOI:** 10.1007/s00417-024-06549-z

**Published:** 2024-06-17

**Authors:** Emilia Felter, Ramin Khoramnia, Maximilian Friedrich, Hyeck-Soo Son, Gerd U. Auffarth, Victor A. Augustin

**Affiliations:** David J. Apple International Laboratory for Ocular Pathology and International Vision Correction Research Centre (IVCRC), Department of Ophthalmology, University of Heidelberg, Heidelberg, INF 400, 69120 Germany

**Keywords:** Keratoconus, Cornea, Biomechanical analysis, Crosslinking, Corvis ST

## Abstract

**Purpose:**

To evaluate the biomechanical and tomographic outcomes of keratoconus patients up to four years after corneal crosslinking (CXL).

**Methods:**

In this longitudinal retrospective-prospective single-center case series, the preoperative tomographic and biomechanical results from 200 keratoconus eyes of 161 patients undergoing CXL were compared to follow-up examinations at three-months, six-months, one-year, two-years, three-years, and four-years after CXL. Primary outcomes included the Corvis Biomechanical Factor (CBiF) and five biomechanical response parameters obtained from the Corvis ST. Tomographically, the Belin-Ambrósio deviation index (BAD-D) and the maximal keratometry (K_max_) measured by the Pentacam were analyzed. Additionally, Corvis E-staging, the thinnest corneal thickness (TCT), and the best-corrected visual acuity (BCVA) were obtained. Primary outcomes were compared using a paired t-test.

**Results:**

The CBiF decreased significantly at the six-month (*p* < 0.001) and one-year (*p* < 0.001) follow-ups when compared to preoperative values. E-staging behaved accordingly to the CBiF. Within the two- to four-year follow-ups, the biomechanical outcomes showed no significant differences when compared to preoperative. Tomographically, the BAD-D increased significantly during the first year after CXL with a maximum at six-months (*p* < 0.001), while K_max_ decreased significantly (*p* < 0.001) and continuously up to four years after CXL. The TCT was lower at all postoperative follow-up visits compared to preoperative, and the BCVA improved.

**Conclusion:**

In the first year after CXL, there was a temporary progression in both the biomechanical CBiF and E-staging, as well as in the tomographic analysis. CXL contributes to the stabilization of both the tomographic and biomechanical properties of the cornea up to four years postoperatively.

## Key message

***What is known:***

• While corneal crosslinking (CXL) is an effective procedure for halting disease progression in keratoconus patients, biomechanical in vivo measurements following CXL have shown conflicting results.

***What is new:***

• Biomechanical and tomographic severity indices CBiF, E-staging, and BAD-D show a temporary worsening within the first year after CXL.

• Post-CXL biomechanical stabilization can be observed up to four years postoperatively when analyzing the corneal response parameters and severity indices.

• Due to corneal thinning following standard protocol CXL, thickness-dependent parameters may not be optimal for the biomechanical and tomographic evaluation after CXL.

## Introduction

Keratoconus is a progressive ectatic disease of the cornea with the highest prevalence during the second and third decades of life [1]. In terms of biomechanics, keratoconus is characterized by reduced corneal elasticity [2], which can be accurately quantified using the Corvis Scheimpflug technology (Corvis ST, Oculus, Wetzlar, Germany) by inducing corneal deformation with an air puff [3]. Recent research has shown that implementing this method improves the precision of diagnosing keratoconus when compared to relying only on tomographic measurements [4]. As the disease naturally progresses, the Corvis ST’s measured biomechanical response parameters deteriorate significantly [5, 6]. These findings suggest the applicability of Corvis ST in evaluating keratoconus progression.

The Corvis Biomechanical Index (CBI) combines various biomechanical response parameters into an index for detecting corneal ectasia [7]. However, due to its binary behavior, it is not suitable for assessing ectasia progression [8]. The newly introduced Corvis Biomechanical Factor (CBiF) is a linear factor based on the CBI that aims to better assess ectasia severity [8]. The CBiF can also be categorized into groups, leading to the E-staging. E-staging has been validated on data from 860 keratoconus corneas [9] to introduce a new standardized grading system, complementing the established tomographic ABCD staging.

Corneal crosslinking (CXL) has been established as a minimally invasive treatment option for progressive keratoconus eyes and was first developed in 2003 in Dresden, Germany [10–12]. CXL induces photochemical cross-links between proteoglycans and collagens, promoting stromal biochemical stiffness and reducing susceptibility to enzymatic digestion [13, 14]. Recent research has demonstrated its safety and effectiveness in halting progression and improving visual acuity [15]. However, more recent studies have yielded mixed results concerning in vivo biomechanical measurements following CXL. While multiple studies utilizing the previously introduced Ocular Response Analyzer (Reichert, Depew, USA) found no discernable differences in corneal biomechanics after CXL [10], a single study employing the more recently developed Corvis ST observed an unexpected progression in the biomechanical severity index CBiF six-months after CXL [16].

The purpose of this study was to further investigate the longitudinal in vivo stabilization in the biomechanical and tomographic properties of the cornea after CXL in keratoconus patients using Corvis ST and Pentacam measurements.

## Materials and methods

This study analyzed 200 eyes from 161 patients longitudinally at the Keratoconus center of the Heidelberg University Hospital between 2021 and 2023. CXL was performed between June 2018 and December 2022 at Heidelberg University Hospital.

The study compared the preoperative tomographic and biomechanical evaluation available from 200 patients using Pentacam and Corvis ST with six follow-up examinations. Follow-up cohorts included 129 patients at three months (2.9 ± 0.8 months), 115 patients at six months (6.4 ± 1.4 months), 94 patients at one year (13.7 ± 2.6 months), 61 patients at two years (2.03 years ± 3.7 months), 33 patients at 3 years (2.95 years ± 3.5 months), and 15 patients at four years (4.03 years ± 4.4 months). Each postoperative cohort was compared to the respective group of eyes preoperatively.

CXL was performed according to the Dresden protocol [12]. First, the eye was anesthetized by topical anesthesia. Then, the central 8–10 mm zone of the epithelium was removed, and a solution comprising 0.1% riboflavin-5-phosphate and 20% dextran was applied to the de-epithelialized surface every two minutes for 30 min. The cornea was then exposed to 370 nm UVA with an irradiance of 3 mW/cm^2^ for 30 min, and the solution was reapplied at 2-minute intervals. After treatment, patients were prescribed ofloxacin antibiotic eye drops, dexamethasone eye drops, and artificial tears, and a soft bandage contact lens.

This study analyzed patients with diagnosed keratoconus and clinical disease progression undergoing CXL with acceptable quality of pre- and postoperative Pentacam and Corvis ST measurements. Keratoconus was diagnosed at a Pentacam BAD-D score of at least 2.6. As outlined by the German Federal Joint Committee of Federal Ministry of Health, the criteria for clinical progression of the disease included an increase in the maximum corneal refractive power by at least 1 diopter (D), an increase in astigmatism by at least 1 D, or a decrease in the base curve of the best-fitting contact lens by at least 0.1 mm [17]. Patients with any underlying conditions other than keratoconus were excluded from the study.

**Fig. 1:**
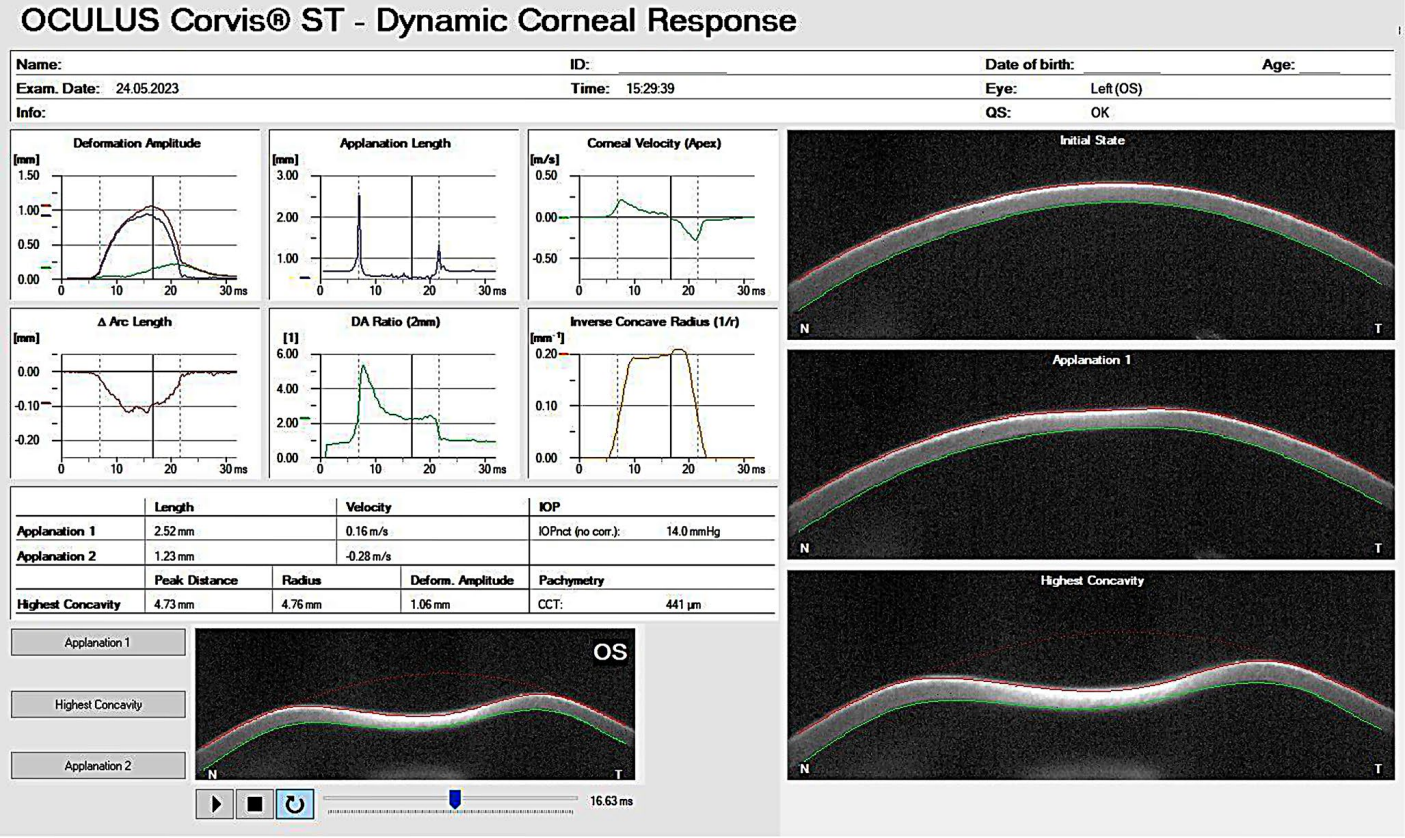
The Corvis ST was utilized to document the dynamic corneal response of a patient one-year following corneal crosslinking. The images on the right (initial state, applanation 1, highest concavity) illustrate the deformation of the cornea during examination. The images on the left display the parameters that describe the characteristics of the corneal deformation throughout the measurement

The primary outcome measures of the study included the CBiF as a biomechanical severity index, as previously described, and the corneal response parameters that form part of the CBiF (Table 1; Fig. 1). The CBiF-derived E-staging classification was included as a secondary endpoint due to its consistent behavior with the CBiF (Table 1). Analyzed response parameters included the deformation amplitude ratio (DA-ratio; the ratio of apical corneal deformation to the deformation at 2 mm away from the apex, Table 1; Fig. 1), the integrated radius (IR; the inverse concave radius at maximum deformation, Table 1; Fig. 1), Ambrósio relational thickness to the horizontal profile (ARTh; the ratio of the thinnest point of the cornea to its increase towards the periphery, Table 1), the stiffness parameter at first applanation (SP-A1; parameter of general rigidity of the cornea, Table 1), and the stress-strain index (SSI; overall stress–strain behavior of the corneal tissue, Table 1). Additionally, the Pentacam-derived Belin-Ambrósio deviation index (BAD-D) as a tomographic severity index as well as changes in the maximal anterior keratometry (K_max_) were selected as primary outcomes. Analysis of the thinnest corneal thickness (TCT) and the best-corrected visual acuity (BCVA) were included as secondary outcome parameters.

**Table 1:** Corvis ST parameters with their definition, formula, unit, normal values in healthy individuals, range (+/- 2 standard deviations) and significance of increasing values

Parameter	Definition	Formula	Unit	Normal values healthy	Range: +/- 2 SD	Higher values indicate
CBiF	Corvis Biomechanical Factor	Combined and linearized index of corneal response parameters	1	≥ 5.94	-	Lower stage of disease
E-Staging	E-Staging	E0 ≥ 5.94 CBiF, -0.58 interval spacing	1	E0	-	Higher stage of disease
DA-ratio	Deformation amplitude ratio at 2 mm	Central deformation/average deformation at 2 mm	1	4.3	3.45–5.15	Softer cornea
IR	Integrated radius	1/ radius of Concave Curvature	mm^− 1^	8.1	6.1–10.1	Softer cornea
ARTh	Ambrósio relational thickness to the horizontal profile	Ratio of the thinnest point of the cornea to its increase towards the periphery	1	440.1	301–579	Thicker cornea
SP-A1	Stiffness parameter at first applanation (A1)	Pressure A1/ corneal displacement A1	1	105.3	72.3-138.3	Stiffer cornea
SSI	Stress-strain-index	Algorithm for material stiffness	1	1.0	0.67–1.33	Stiffer cornea

The data were analyzed utilizing SPSS Statistics, version 29 (IBM, Armonk, USA). Initially, outliers and normal distribution of data were assessed for comparing cases with less than 30 counts. This was performed utilizing exploratory data analysis and the Shapiro-Wilk test, under the assumption of normal distribution if *p* > 0.05. Outliers that have a standard deviation greater than three were removed from subsequent analysis. Preoperative measurements were compared to each follow-up time point using a two-tailed paired t-test for normally distributed data. For non-normally distributed data, the Wilcoxon signed-rank test was employed for comparison. To account for multiple comparisons, a Bonferroni correction was applied. Given eight primary outcome parameters and six comparisons per parameter, the resulting alpha was calculated as α < 0.00104 (rounded: α < 0.001). Patients who had missing follow-up data were excluded on a case-by-case basis for the defined follow-up time points. The necessary sample size for the main outcome measures was determined using d = 0.5 (estimated effect size), α = 0.00104 significance level, and power of *p* = 0.8 with a sample size of 74.

## Results

Of the 161 patients included in this study, 122 were male (75.8%), and the ratio of right eyes (49.5%) was comparable to that of left eyes (50.5%). The mean age at CXL was 24.6 (± 7.4) years.

**Fig. 2:**
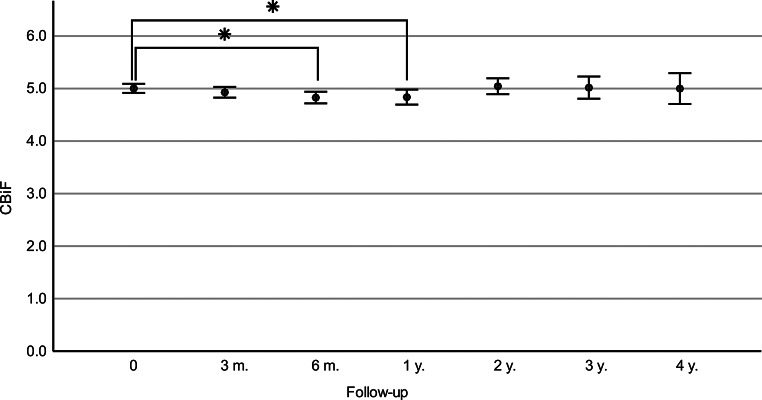
Means and standard deviations of Corvis Biomechanical Factor (CBiF) preoperatively (0) and at the follow-up visits (m.=months, y.=years). Comparison of mean CBiF preoperatively to follow-up reveals statistically significant differences (*=*p* < 0.001) at six-months and one-year following CXL

The CBiF significantly decreased at the six-month and one-year follow-ups in comparison to preoperative measurements (Fig. 2; Table 2). Subsequently, the CBiF measurements approached preoperative values once again after two years. Accordingly, the derived biomechanical E-staging showed a temporary increase at six-months and one-year follow-ups to then decrease to preoperative levels after 2 years and beyond (Table 2).

**Table 2:** Mean differences and standard deviation of biomechanical and tomographic parameters at the respective follow-up compared to the preoperative value

Group	Pre-operative value	Difference to preoperative value at					
		3 months	6 months	1 year	2 years	3 years	4 years
Count (*n*)	200	129	115	94	61	33	15
CBiF	5.00±0.6	-0.08±0.3**	-0.17±0.3***	-0.15±0.4***	-0.11±0.4*	-0.07±0.4	+ 0.07±0.3
E-Staging	2.57±1.1	+ 0.14±0.6**	+ 0.28±0.5***	+ 0.26±0.6***	+ 0.21±0.7*	+ 0.14±0.7	-0.11±0.5
DA-ratio	5.88±4.9	-0.19±0.7**	-0.22±0.8**	-0.11±0.7	-0.20±0.5**	-0.22±0.5	-0.43±0.4***
IR (mm^− 1^)	10.5±2.5	-0.44±1.5**	-0.28±1.6	+ 0.17±1.6	-0.04±1.2	+ 0.23±1.7	-0.05±1.2
ARTh	255.4±122	-49.0±80***	-63.4±85***	-76.2±94***	-81.3±100***	-78.3±75***	-62.3±61**
SP-A1	63.9±19	-1.9±11	-3.1±11**	-0.6±13	+ 1.4±12	+ 4.7±14	+ 8.7±17
SSI	0.90±0.25	+ 0.07±0.17***	+ 0.07±0.17***	+ 0.03±0.20	+ 0.04±0.17	±0.00±0.17	+ 0.04±0.20
BAD-D (SD)	8.64±4.5	+ 0.48±1.2***	+ 0.61±1.1***	+ 0.59±1.2***	+ 0.29±1.1	+ 0.54±2.2	+ 0.13±2.6
K_max_ (D)	56.0±8.3	-0.2±1.6	-1.5±1.7***	-2.2±2.0***	-2.1±2.3***	-3.6±2.7***	-3.6±2.2***
TCT (µm)	466.5±38	-20.1±17***	-24.8±25***	-26.0±24***	-25.0±31***	-32.1±28***	-24.7±18***
BCVA (logMAR)	0.27±0.25	+ 0.03±0.16	-0.03±0.26	-0.07±0.22*	-0.10±0.24*	-0.10±0.18	-0.16±0.19*

*p < 0.05 **p < 0.01 ***p < 0.001. *CBiF* corvis biomechanical factor, *E-Staging* corvis e-staging, *DA-ratio* deformation amplitude ratio, *IR* integrated radius in mm^− 1^, *ARTh* Ambrósio relational thickness to the horizontal profile, *SP-A1* stiffness parameter at first applanation, *SSI stress*-strain-index, *BAD-D* Belin/Ambrósio Deviation Index in SD, *K*_*max*_ maximal keratometry in D, *TCT* thinnest corneal thickness in µm, *BCVA* best-corrected visual acuity in logMAR. Bold: *p* < 0.001

**Fig. 3:**
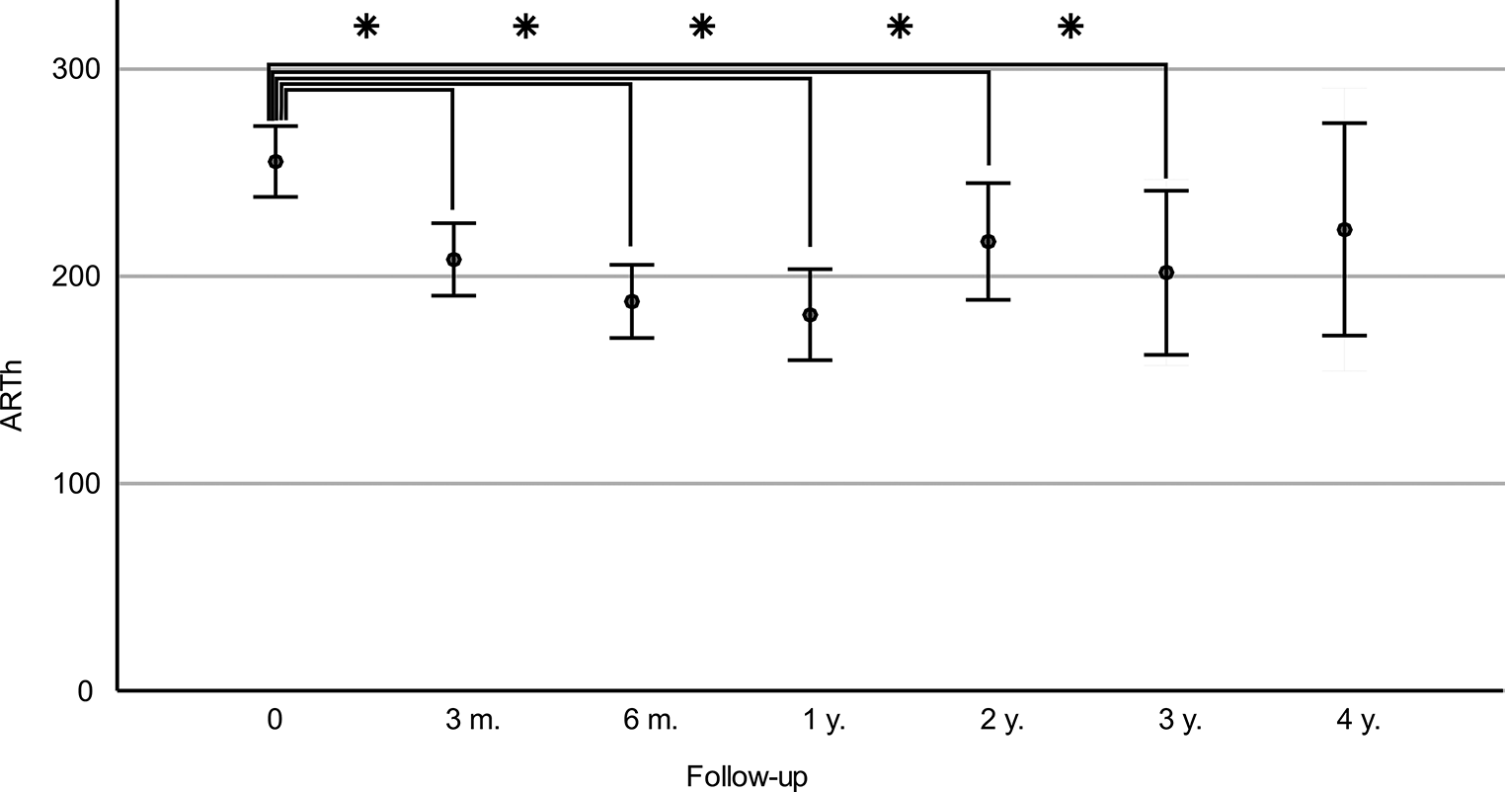
Means and standard deviations of preoperative Ambrósio relational thickness to the horizontal profile (ARTh) and at follow-up visits. The comparison of mean ARTh before surgery (0) to follow-ups (m.=months, y.=years) shows significant differences (*=*p* < 0.001) at three-months, six-months, one-year, two-years, and three-years after CXL

**Fig. 4:**
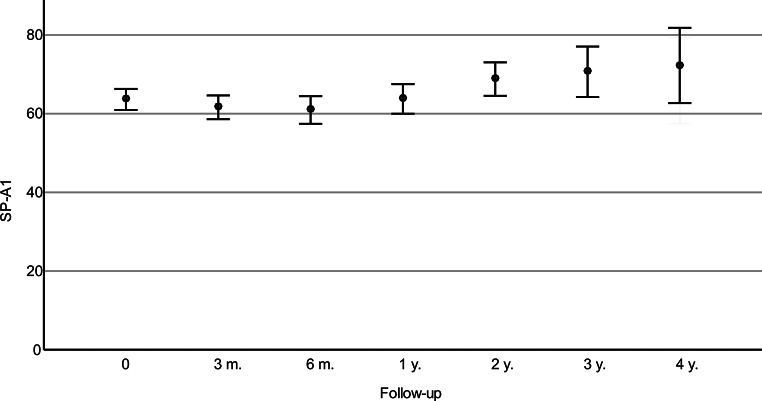
Means and standard deviations of preoperative stiffness parameter at first applanation (SP-A1) and SP-A1 at follow-up visits. The comparison of mean SP-A1 from before (0) the operation to follow-up visits (m.=months, y.=years) indicates a statistically insignificant growth

The DA-ratio demonstrated a decrease with statistically significant changes observed at four years-postoperatively (Table 2), while the IR did not exhibit statistically significant differences from the preoperative values after Bonferroni correction at any follow-up examination (Table 2). ARTh was significantly lower than the pre-CXL value at any follow-up point in time between three months and three years after CXL (Fig. 3; Table 2). The SP-A1 decreased until one year after the surgery and then consistently increased, though not significantly (Fig. 4; Table 2). The SSI saw a significant increase during the first year after CXL and then decreased to preoperative levels (Table 2).

The K_max_ obtained from the Pentacam consistently and significantly decreased during every follow-up visit from six-months to four-years after CXL (Table 2). During the same follow-up period, BAD-D increased significantly from the three-months to one-year visit before returning to preoperative levels (Table 2). Also, the TCT significantly decreased after surgery during all follow-up visits when compared to preoperative measurements (Table 2). The mean BCVA improved over time, reaching − 0.16 logMAR at four years post-CXL (Table 2).

## Discussion

This study aimed to determine whether biomechanical stabilization can be measured following CXL in accordance with the long-known tomographic stabilization. Within the first year after CXL, we observed a significant decrease in CBiF and an increase in E-Staging. Thereafter, the values remained consistent with preoperative levels from two to four years postoperatively. Biomechanical progression of keratoconus corresponds to a decrease in CBiF and an according increase in E-staging [9]. Therefore, the findings suggest a biomechanical progression of disease severity during the initial year after surgery followed by biomechanical stabilization. BAD-D, a tomographic index of ectasia detection, showed an increase over a similar period after CXL with significant changes at three-months, six-months, and one-year postoperatively. However, after two years and more, the CBiF, E-staging, and BAD-D showed values similar to those before surgery. Another study conducted at the Homburg Keratoconus Center on 22 corneas six months after CXL showed the same phenomenon with a biomechanical and tomographic increase in CBiF, E-Staging, and BAD-D, but no significant differences after one and two years [16]. These findings, which are similar to those of our study, suggest biomechanical stabilization *after* the first postoperative year.

K_max_, a parameter that is also used for disease classification [1], however, decreased continuously and immediately after CXL. The measurements exhibited an average decrease of 1.5 D after three months and 3.6 D after three years when compared to pre-treatment measurements. This suggests that actual disease progression may not occur within the initial year. Instead, there appears to be an ongoing stabilization of the cornea and a reduction in corneal protrusion immediately following CXL. The significant reduction in K_max_ after CXL has been reported in multiple studies, accompanied by a slight improvement in visual acuity [18–20]. Contrastingly, a comprehensive meta-analysis of 11,529 eyes revealed a significant K_max_ increase within one year amongst progressive keratoconus eyes left untreated [21]. This finding underlines the importance of CXL treatment to hinder keratoconus progression. Also, our study demonstrated an enhancement of BCVA as shown by an improvement of up to -0.16 logMAR after a four-year follow-up period.

The biomechanical severity parameter CBiF, the derived E-staging, and the tomographic BAD-D index are strongly influenced by corneal thickness measurements [8, 22]. In our study, the TCT significantly decreased at all postoperative follow-up time points. A systematic meta-analysis of six studies, including 261 eyes, also found a significant decrease in TCT after the standard CXL protocol as utilized in this study [20]. Other studies, using different CXL protocols found measurement artifacts due to stromal changes after CXL within the first postoperative year, leading to an underestimation of the corneal thickness measured by Scheimpflug technology [16, 23]. Our study suggests an actual decrease in corneal thickness as TCT measurements remained at the same lower level even four years after CXL. Nevertheless, the decrease in corneal thickness after CXL confounds the comparison of corneal response parameters and severity indices. A modified biomechanical index, such as that proposed in a publication by Steinberg et al. [24] without ARTh and therefore taking corneal thickness less into account, shows slightly reduced sensitivity in detecting keratoconus [25]. Nevertheless, it may still be a more accurate option in determining improvements in biomechanical properties after standard protocol CXL.

Regarding the biomechanical response parameters used to calculate CBiF and E-staging, the parameter most affected by TCT is ARTh [7]. It is expected for ARTh to be significantly decreased at every post-operative follow-up visit up to three years post-CXL, given that TCT was significantly lower at each visit. These results correspond with those of Flockerzi et al., who similarly documented a notable decrease in ARTh during every subsequent assessment within a two-year observation period [16].

The SP-A1 is an interesting corneal response parameter for assessing corneal biomechanics due to its direct relation to corneal stiffness, with greater values corresponding to heightened stiffness [7]. The present study documented a decrease in SP-A1 following surgery, with its lowest value at six-months after CXL. Thereafter, the SP-A1 showed continuously increasing values at each follow-up visit up to four years post-CXL. Other studies that investigated this parameter showed similar results. Two recent studies respectively examining 21 corneas two years after accelerated CXL have shown a significant increase in SP-A1 [16, 26]. Additionally, another study found a significant increase in SP-A1 on 67 corneas after just six months [27]. The initial decrease in corneal stiffness observed in this study may be attributed to its correlation with corneal thickness [28]. Other treatment protocols as used in the above-mentioned studies may have less effect on corneal thickness [20], leading to more pronounced changes in SP-A1. However, the comparable trend of a continuous increase in this parameter supports the notion of a gradual postoperative stiffening of the cornea up to four years after CXL. While IR also showed no significant differences from pre- to postoperative, this can also be interpreted as a stabilization due to its deterioration in untreated progressive corneas [5].

The DA-ratio is another biomechanical parameter indicative of corneal stiffness. In our study, the parameter consistently decreased and reached statistically significant changes four years after surgery, supporting the hypothesis of a gradual and continuous corneal stiffening post-CXL. This argument stems from lower parameter values indicating higher resistance to deformation [7]. Two other studies also reported a significantly decreased DA-ratio among 21 corneas two years after CXL [26] and 67 corneas six months after CXL [27].

The SSI is a newly developed parameter for assessing the biomechanical behavior of the cornea in vivo, with no correlation to corneal thickness or intraocular pressure [29]. Higher SSI values indicate greater tissue stiffness [29]. Interestingly, the initial two follow-ups at the three-month and six-month post-CXL periods recorded higher SSI values, while no significant alterations to preoperative measurements were observed thereafter. This suggests an immediate increase in corneal stiffness and stabilization with no significant difference after this period. The initial increase in SSI contrasts with the first-year changes observed in CBiF, E-staging, BAD-D, and SP-A1, possibly due to its independence from corneal thickness. While the SSI has been reported to deteriorate in untreated progressive eyes [5, 6], comparable values to preoperatively support biomechanical stabilization. However, further studies are needed to investigate this parameter in the context of CXL.

A limitation of this study is the high rate of participants who dropped out during later follow-up periods. Consequently, the two- to four-year results should be interpreted with caution as the initially calculated effect size was not met at these visits. Furthermore, a very strict significance level was imposed due to the multiple comparisons. However, this study analyzed the biomechanical stabilization over a long follow-up compared to other studies [16, 26, 27] and offers valuable insights into corneal stiffening up to four years after CXL. Further research comparing our data to a healthy control group or keratoconus patients without disease progression may be beneficial in enhancing the interpretation of our analysis.

In conclusion, this study provides empirical evidence of biomechanical in vivo stabilization up to four years post-CXL. The biomechanical severity index CBiF as well as certain biomechanical response parameters such as the ARTh appear to be affected by corneal thinning following standard protocol CXL and, hence, may not be optimal for evaluating the efficacy of CXL at least in the early postoperative period. However, the biomechanical results of the corneal response parameters SP-A1, DA-ratio, IR, and SSI showed no deterioration when compared to preoperatively. The equalization of CBiF to preoperative levels one year after CXL up to four years after CXL, despite the continued lower TCT measurements, suggests long-term corneal biomechanical stabilization.
